# The value of lateral flow urine lipoarabinomannan assay and empirical treatment in Xpert MTB/RIF ultra negative patients with presumptive TB: a prospective cohort study

**DOI:** 10.1038/s41598-021-04090-1

**Published:** 2021-12-24

**Authors:** Wakjira Kebede, Gemeda Abebe, Esayas Kebede Gudina, Annelies Van Rie

**Affiliations:** 1 Department of Epidemiology and Social Medicine, University of Antwerp, Antwerp, Belgium; 2 Mycobacteriology Research Center, Jimma University, Jimma, Ethiopia; 3 School of Medical Laboratory Science, Jimma University, Jimma, Ethiopia; 4 Department of Internal Medicine, Jimma University, Jimma, Ethiopia

**Keywords:** Microbiology, Medical research

## Abstract

The value of Lateral Flow urine Lipoarabinomannan (LF-LAM) assay and the role of empiric tuberculosis (TB) treatment in the era of the highly sensitive Xpert MTB/RIF Ultra (Xpert Ultra) assay is unclear. This study aimed to assess the additional yield of LF-LAM assay when used in combination with Xpert Ultra, and the role of empiric TB treatment when Xpert Ultra used as the initial diagnostic in presumptive TB cases admitted to a tertiary hospital in Ethiopia. We performed a secondary analysis of a cohort of hospitalized Xpert MTB/RIF-negative patients. Sputum samples were examined for *Mycobacterium tuberculosis* by culture and Xpert Ultra. In HIV positive and severely ill patients, a urine sample was collected for the LF-LAM assay. Treatment outcome was assessed six months after enrollment. Logistic regression was used to identify factors predictive of deaths among Xpert Ultra negative patients. The Xpert Ultra assay diagnosed 31 of the 35 culture positive among the 250 hospitalized Xpert MTB/RIF-negative participants. The LF-LAM assay did not identify any case not detected by Xpert Ultra among the 52 (21.4%) participants living with HIV and the 16 patients with severe disease. Among Xpert Ultra negative patients, those who received empirical TB treatment had a similar odds of death (aOR 0.74, 95% CI: 0.1–2.7) as those not started on TB treatment. Low body mass index (≤ 18.5 kg/m^2^) was the only significant predictor of death in Xpert Ultra negative patients (aOR 4. 0, 95% CI: 1.08–14.6). In this prospective cohort, LF-LAM did not improve the diagnostic yield when used in combination with Xpert Ultra. Empiric TB treatment for Xpert Ultra negative presumptive TB cases was not associated with death at six months. Future studies in diverse settings should be to determine the optimal management of Xpert Ultra negative patients.

## Introduction

Tuberculosis (TB) is a chronic infectious disease caused by *Mycobacterium tuberculosis* (MTB) complex^1^. With an estimated 10 million active TB cases, of which 10% are co-infected with HIV, and 1.3 million deaths in 2019, TB remains a public health problem worldwide^2^. In 2019, Ethiopia had the 10th highest incidence of TB globally, with 114, 233 new cases and an incidence rate of 151 cases per 100,000 people^2,3^.

Even though the World Health Organization (WHO) endorsed the molecular Xpert MTB/RIF assay almost a decade ago^4^, many primary care clinics in high burden countries still rely on smear microscopy as the initial diagnostic test. Because of the low sensitivity of smear microscopy, smear negative patients with TB symptoms are often empirically started on TB treatment^5^. Even when the Xpert MTB/RIF assay is available, empirical treatment remains highly prevalent in high TB burden countries^6^. The use of empirical treatment can be justified in these settings given the suboptimal performance of the Xpert assay, especially among smear negative individuals (sensitivity of 67%) and in people living with HIV (sensitivity of 80%)^7,8^.

In 2017, the WHO recommended replacing Xpert MTB/RIF by Xpert MTB/RIF Ultra (Xpert Ultra) as the initial diagnostic test because of its superior performance in smear negative patients and patients living with HIV^9^. A meta-analysis of 19 studies found an overall pooled sensitivity of 84% the Xpert Ultra compared to 69% for Xpert MTB/RIF^10^. In smear-negative TB cases, Xpert Ultra was 17% more sensitive compared to Xpert MTB/RIF; in people living with HIV Xpert Ultra was 13% more sensitive^11–13^. Whether empiric TB treatment is still justified in high TB burden settings when Xpert Ultra is used as the initial diagnostic has not yet been investigated.

The lateral flow urine lipoarabinomannan assay (LF-LAM) is another TB diagnostic tool that was recently endorsed but is not yet commonly used in high TB burden countries^14^. In inpatient settings, LF-LAM can assist in the diagnosis of TB in people living with HIV. Among people living with HIV who present with signs and symptoms of TB to an inpatient setting, the pooled sensitivity of LF-LAM was 52% (40–64%) and pooled specificity 87% (78–93%). When used in combination with other tests, LF-LAM can improve the diagnostic yield by 14% (compared to Xpert MTB/RIF alone) or 36.6% (compared to clinical sign and symptoms alone)^15,16^. It has also been shown that the use of LF-LAM can improve the survival of patients hospitalized with advanced HIV disease [pooled risk ratio for mortality 0.85 (0.76–0.94)]^14,17,18^. The yield of LF-LAM when used in combination with Xpert Ultra has not yet been assessed.

In this study, we aimed to assess the additional yield of LF-LAM assay when used in combination with Xpert Ultra, and the role of empiric TB treatment in Xpert Ultra negative patients with symptoms of TB admitted to a tertiary hospital in Ethiopia.

## Results

### Characteristics of the study population

**Figure 1:**
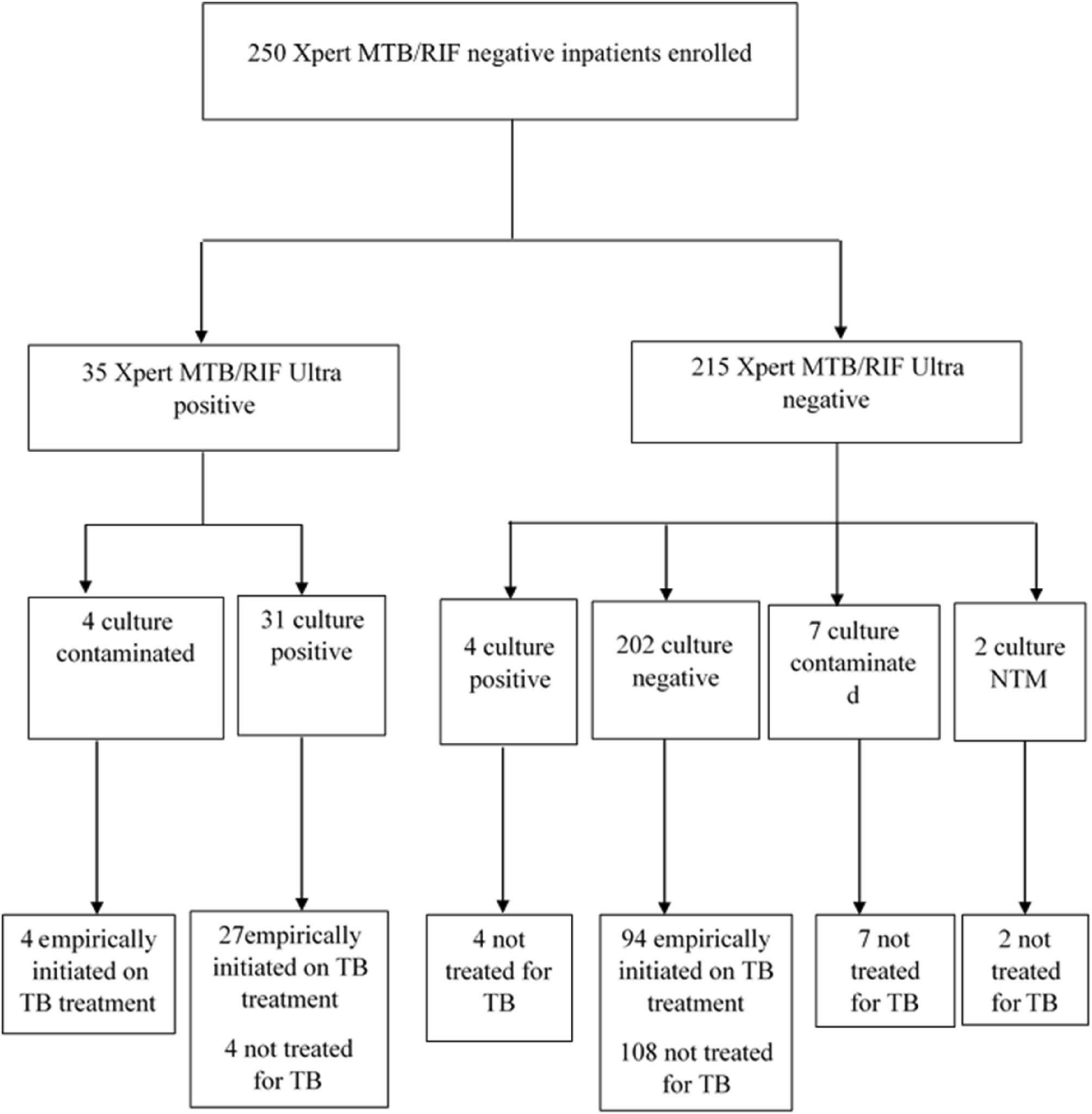
Patient enrollment flowchart showing the number of patients enrolled, Xpert Ultra result, culture result and treatment status distribution. TB, tuberculosis; MTB, *Mycobacterium tuberculosis*; NTM, non-tuberculosis mycobacteria.

All 250 patients admitted to the hospital with signs or symptoms of TB who were enrolled in the parent study were included in the secondary analysis. By design, all study participants had a negative Xpert MTB/RIF result (Fig. 1). The majority were 40 years of age or younger (144/250, 57.6%), female (138/250, 55.2%), and rural residents (154/250, 57.6%). Almost all patients (232/250, 92.8%) had already visited another health facilities for similar complaints before their current hospitalization. HIV status was documented for 243 (97.2%) participants of which 21.4% were positive (Table 1). All participants had symptoms of current cough, fever, night sweat and shortness of breath; 165 (66%) had a cough for ≥ 14 days, 45 (18%) had a history of previous TB treatment and (93/250, 37.6%) had a chest X-ray findings typical or compatible with TB.

**Table 1:** Demographic and clinical characteristics of 250 Xpert MTB/RIF-negative patients hospitalized at Jimma University Medical Center, Oromia, Ethiopia.

Characteristics	Category	All	Xpert ultra test results	
			Positive	Negative
Patients n		250	35	215
Age	≤ 40 years	144 (57.6)	29 (82.9)	115 (53.5)
	> 40 years	106 (42.4)	6 (17.1)	100 (46.5)
Sex	Female	138 (55.2)	22 (62.9)	116 (54.0))
	Male	112 (44.8)	13 (37.1)	99 (46.0)
Educational status	Illiterate	116 (46.4)	8 (22.9)	108 (50.2)
	Primary level	51 (20.4)	8 (22.9)	43 (20.0)
	Secondary & above	83 (33.2)	19 (54.2)	64 (29.8)
Residence	Urban	106 (42.4)	15 (42.9)	91 (42.3)
	Rural	144 (57.6)	20 (57.1)	124 (57.7)
Health facilities visited prior to hospital admission	Local government hospitals	126 (50.4)	9 (25.7)	117 (54.4)
	Primary care center	48 (19.2)	10 (28.6)	38 (17.7)
	Private Clinic	58 (23.2)	13 (37.1)	45 (20.9)
	None	18 (7.2)	3 (8.6)	15 (7.0)
Body mass index	≤ 18.5 kg/m^2^	84 (33.6)	22 (62.9)	62 (28.8)
	> 18.5 kg/m^2^	166 (66.4)	13 (37.1)	153 (71.2)
HIV status	Negative	191 (76.4)	24 (68.8)	167 (77.7)
	Positive	52 (20.8)	11 (31.4)	41 (19.1)
	Unknown	7 (2.8)	0 (0.0)	7 (3.2)
CD4 count (n = 52)	≤ 200 cells/ml	24 (46.2)	10 (90.9)	14 (26.9)
	> 200 cells/ml	28 (53.8)	1 (9.1)	27 (73.1)
History of TB treatment	No	205 (82.0)	27 (77.1)	178 (82.8)
	Yes	45 (18.0)	8 (22.9)	37 (17.2)
Clinical severity	Non-severe	232 (92.8)	30 (85.7)	202 (94.0)
	Severe	18 (7.2)	5 (13.9)	13 (6.0)
Weight loss	No	111 (44.4)	9 (25.7)	102 (47.4)
	Yes	139 (55.6)	26 (74.3)	113 (52.6)
Pleuritic chest pain	No	82 (32.8)	5 (14.3)	77 (35.8)
	Yes	168 (67.2)	30 (85.7)	138 (64.2)
Loss of appetite	No	49 (19.6)	2 (5.7)	47 (21.9)
	Yes	201 (80.4)	33 (94.3)	168 (78.1)
Duration of cough	< 14 days	85 (34.0)	5 (14.3)	80 (37.2)
	≥ 14 days	165 (66.0)	30 (85.7)	135 (62.8)
Duration of fever	< 14 days	137 (54.8)	17 (48.6)	120 (55.8)
	≥ 14 days	113 (45.2)	18 (51.4)	95 (44.2)
Duration of night sweats	< 14 days	130 (52.0)	18 (51.4)	112 (52.1)
	≥ 14 days	120 (48.0)	17 (48.6)	103 (47.9)
Duration of shortness of breath	< 14 days	123 (49.2)	10 (28.6)	113 (52.6)
	≥ 14 days	127 (50.8)	25 (71.4)	102 (47.4)

Data are presented as n (%), unless otherwise stated.

### Results of *Mtb* culture and Xpert Ultra assay

Of the 250 cultures, 35 (14.0%) were positive for *Mtb* complex and 2 (0.8%) for non-tuberculosis mycobacteria (NTM), 202/250 (80.8%) were *Mtb* culture negative and 11/250 (4.4%) were contaminated (Table 2).

**Table 2:** Xpert Ultra and LAM test results compared with mycobacterial culture for the diagnosis of pulmonary TB among Xpert MTB/RIF-negative patients hospitalized at Jimma University Medical Center, Oromia, Ethiopia.

	Mycobacterial culture results				
	LJ or MGIT positive	LJ or MGIT negative	LJ or MGIT contaminated	NTM	Total
**Xpert MTB/RIF Ultra**					
*Mtb* detected*	27 (77.2)	0 (0.0)	4 (36.4)	0 (0.0)	31 (12.4)
*Mtb* Trace	4 (11.4)	0 (0.0)	0 (0.0)	0 (0.0)	4 (1.6)
*Mtb* not detected	4 (11.4)	202 (100)	7 (63.6)	2 (100)	211 (84.4)
Total	35	202	11	2	250
**LF-LAM in HIV positive participants**					
Positive	6 (60)	0 (0.0)	0 (0.0)	0 (0.0)	6 (11.5)
Negative	4 (40)	39 (100)	2 (100)	1 (100)	46 (88.5)
Total	10	39	2	1	52

Data are presented as n (%), unless otherwise stated.

*All were sensitive to rifampicin.

*Mtb* Mycobacterium tuberculosis; *NTM* Non tuberculosis mycobacteria, *LJ* Lowenstein Jensen medium, *MGIT* Mycobacteria Growth Indicator Tube, *TB* Tuberculosis, *LF-LAM* Lateral Flow urine Lipoarabinomannan.

The Xpert Ultra assay was positive for *Mtb* complex in 35/250 (14%), of which 4 (11.4%) had a trace call. The Xpert Ultra result was negative in 215 (86.0%) participants, including four samples that were repeated because of an error result on the initial test (Table 2). The Xpert Ultra assay was positive in 4 patients with a contaminated culture. One rifampicin resistant strain identified by LPA was not identified as rifampicin resistant by Xpert Ultra.

Participants whose sputum cultures were contaminated (n = 11) or positive for NTM (n = 2) were excluded from this analysis. Of the remaining 237 participants, 35 (14.8%) had culture-confirmed TB. The Xpert Ultra assay detected 31 (88.6%) of the 35 culture-confirmed cases. The 4 culture-confirmed cases that were missed by Xpert Ultra were HIV negative. The assay was positive in 4 (36.4%) of the 11culture contaminated and was negative in all 202 culture-negative participants. The 4 Xpert Ultra positive culture-contaminated cases had very low mycobacterial burden as demonstrated by a high cycle threshold (Ct) value (Ct value of the lowest probe ≥ 29.2, Supplemental Table), were all HIV negative, and none of these cases had a history of prior TB treatment.

Of the eight Xpert MTB/RIF negative patients not started on empiric treatment, four were started on TB treatment based on a positive culture and four based on a positive Xpert Ultra.

### The yield of LF-LAM test

**Figure 2:**
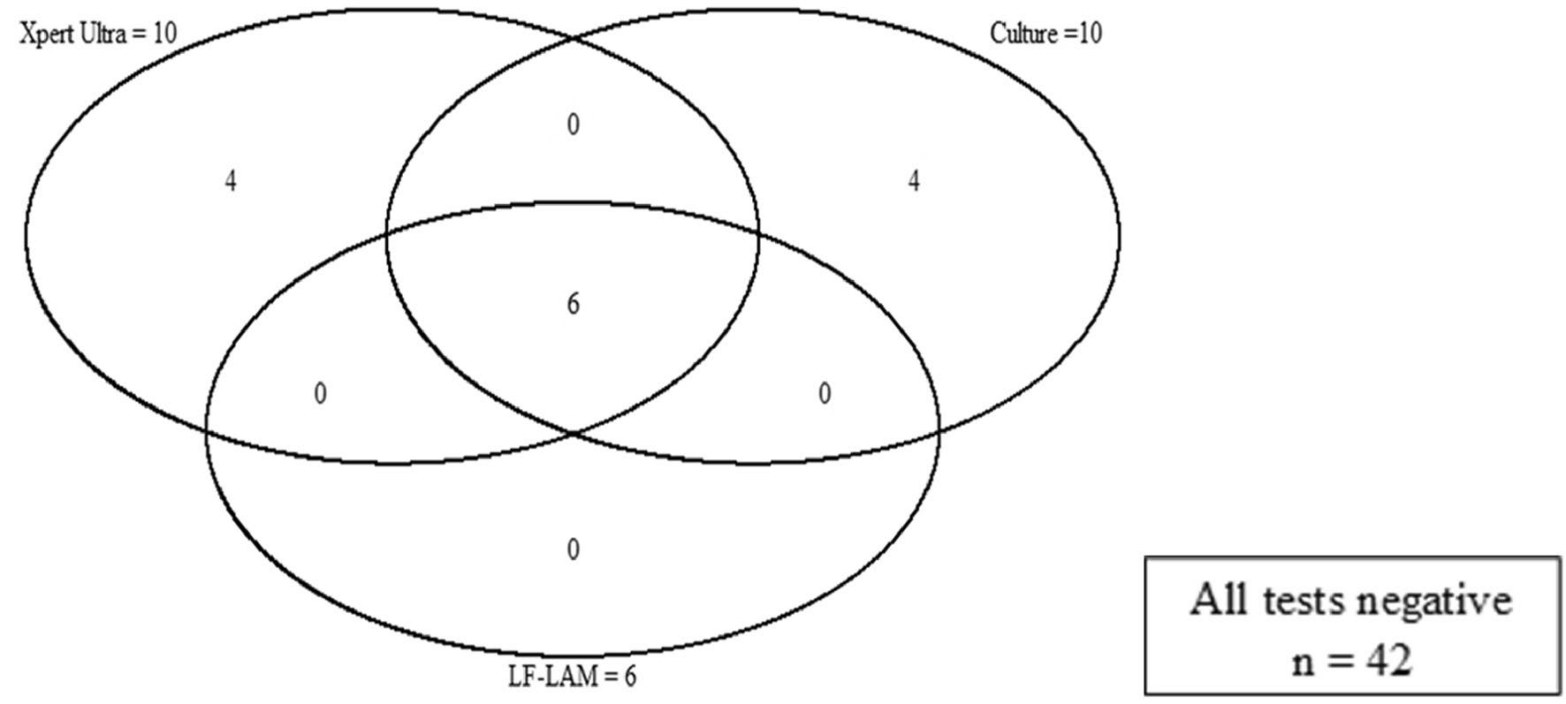
Sputum culture, Xpert Ultra and urine LF-LAM results in 52 HIV-positive Xpert MTB/RIF-negative hospitalized patients. Xpert Ultra, Xpert MTB/RIF Ultra; LF-LAM, Lateral Flow Urine Lipoarabinomannan; HIV, Human Immunodeficiency virus.

Among 52 HIV positive participants assessed by LF-LAM, 6 (11.5%) LF-LAM tests were positive (Fig. 2). Six of the 10 (60.0%) culture positive Xpert Ultra positive cases were detected by LF-LAM. In addition, 3 of 16 (19%) of severely ill HIV negative patients assessed by LF-LAM had a positive LF-LAM result, including two Xpert Ultra positive but culture negative participants. None of the LF-LAM positive cases were Xpert Ultra negative. As such, the LF-LAM assay did not increase the diagnostic yield when used in combination with Xpert Ultra.

### Role of empiric TB treatment in Xpert Ultra negative patients

Of the 215 (86%) Xpert Ultra negative participants, 94 (43.7%) were diagnosed clinically and empirically started on TB treatment, and 121 (56.3%) were clinically considered not to have TB and were not initiated on TB treatment. Of the 94 Xpert Ultra negative participants started on empiric TB treatment, 90 (95.7%) were alive and 4 (4.3%) had died in the 6 months following assessment. Of the 121 Xpert Ultra negative patients not started on TB treatment, 115 (95.0%) were alive and 6 (5.0%) had died in the 6 months following assessment (Table 3). Among Xpert Ultra negative patients, empirical TB treatment had similar odds of death but the small sample size resulted in wide 95% confidence intervals (aOR 0.74, 95% CI 0.1–2.7).

**Table 3:** Role of empiric TB treatment in the Xpert Ultra era among Xpert MTB/RIF-presumptive TB cases hospitalized at Jimma University Medical Center, Oromia, Ethiopia.

TB treatment status & outcomes	All patients	Xpert Ultra test results	
		Positive	Negative
Empirically initiated on TB treatment	125/250 (50.0)	31/35 (86.6)	94/215 (43.7)
**Outcome**			
Alive	118/125 (94.4)	28/31 (90.3)	90/94 (95.7)
Died	7/125 (5.6)	3/31 (9.7)	4/94 (4.3)
Not initiated on TB treatment	125/250 (50.0)	4/35 (11.4)	121/215 (56.3)
**Outcome**			
Alive	119/125 (95.2)	4/4^**#**^ (100.0)	115/121 (95.0)
Died	6/125 (4.8)	0/4 (0.0)	6/121 (5.0)
Total	250	35	215

Data are presented as n (%), unless otherwise stated.

^#^Both liquid (MGIT) and solid (LJ) cultures were contaminated, but 2 of the 4 cases were LF-LAM positive.

### Predictor of 6-month mortality among Xpert Ultra negative patients

In regression analysis, it was found that underweight or body mass index (BMI) ≤ 18.5 kg/m2 was significant predictor of mortality among Xpert Ultra negative patients (aOR 4.0, 95% CI: 1.08–14.6) (Table 4).

**Table 4:** Patient characteristics associated with mortality among Xpert Ultra negative patients hospitalized at Jimma University Medical Center, Oromia, Ethiopia.

Characteristics	Category	Outcome		Crude odds ratio (95% CI)	Adjusted odds ratio (95% CI)
		Died	Alive		
Patients n		10	205		
Age	≤ 40 years	5(50.0)	108(52.7)	Ref	
	> 40 years	5 (50.0)	97 (47.3)	1.11 (0.3–4.1)	
Sex	Female	5 (50.0)	112 (54.6)	Ref	
	Male	5 (50.0)	93 (45.4)	1.2 (0.3–4.4)	
Educational status	Secondary & above	1 (10.0)	43 (21.0)	Ref	
	Primary level	3 (30.0)	60 (29.3)	2.1 (0.26–44.2)	
	Illiterate	6 (60.0)	102 (49.8)	2.5 (0.41–48.5)	
Residence	Urban	5 (50.0)	87 (42.4)	Ref	
	Rural	5 (50.0)	118 (57.7)	0.73 (0.2–2.7)	
Clinical severity	Non-severe	9 (90.0)	190 (94.1)	Ref	Ref
	Severe	1 (10.0)	12 (5.9)	1.7 (0.19–10.7)	0.2 (0.03–5.8)
Body mass index	> 18.5 kg/m^2^	4 (40.0)	150 (73.2)	Ref	Ref
	≤ 18.5 kg/m^2^	6 (60.0)	55 (26.8)	4.0 (1.12–16.5)	4.0 (1.08–14.6)*
HIV status^a^	Negative	6 (60.0)	160 (78.0)	Ref	Ref
	Positive	4 (40.0)	38 (18.5)	2.8 (0.7–10.3)	2.1 (0.4–8.7)
Weight loss	No	4 (40.0)	99 (48.3)	Ref	Ref
	Yes	6 (60.0)	106 (51.7)	1.4 (0.38–5.61)	0.6 (0.1–2.7)
Loss of appetite	No	1 (10.0)	46 (22.4)	Ref	Ref
	Yes	9 (90.0)	159 (77.6)	2.6 (0.47–48.6)	0.3 (0.02–2.3)
Pleuritic chest pain	No	4 (40.0)	74 (36.1)	Ref	Ref
	yes	6 (60.0)	131 (63.9)	0.84 (0.23–3.4)	1.2 (0.2–6.7)
History of TB treatment	No	9 (90.0)	169 (82.4) 36	Ref	
	Yes	1(10.0)	(17.6)	0.5 (0.02–2.9)	
Duration of fever	< 14 days	6 (60.0)	115 (56.1)	Ref	Ref
	≥ 14 days	4 (40.0)	90 (43.9)	0.85 (0.21–3.07)	1.1 (0.2–6.0)
Duration of cough	< 14 days	4 (40.0)	77 (37.6)	Ref	Ref
	≥ 14 days	6 (60.0)	128 (62.4)	0.9 (0.24–3.62)	0.8 (0.1–3.9)
Duration of night sweats	< 14 days ≥ 14 days	6 (60.0) 4 (40.0)	107 (52.2) 98 (47.8)	Ref 0.72 (0.18–2.62)	Ref 2.0 (0.4–11.7)
Duration of shortness of breath	< 14 days ≥ 14 days	5 (50.0) 5 (50.0)	109 (53.2) 96 (46.8)	Ref 1.13 (0.30–4.19)	Ref 0.5 (0.09–2.7)
Culture results	Negative	9 (90.0)	202 (98.5)	Ref	
	Positive	1 (10.0)	3 (1.5)	7.48 (0.35–65.5)	
Empiric treatment	No	6 (60.0)	115 (56.1)	Ref	Ref
	Yes	4 (40.0)	90 (43.9)	0.85 (0.21–3.07)	0.74 (0.1–2.7)

Data are presented as n (%).

Significant values are in bold.

*CI* confidence interval.

*p value = 0.041.

^a^7 missing HIV test results.

## Discussion

In this prospective cohort study, we confirmed the high sensitivity of Xpert Ultra among cases of presumptive TB, as 89% (31 of the 35) of the culture positive cases missed by Xpert MTB/RIF were positive on Xpert Ultra. We demonstrated that the use of empirical treatment may no longer be justified in Xpert Ultra negative presumptive pulmonary TB patients as it does not impact the odds of death, and that LF-LAM does not increase the diagnostic yield among HIV positive and severely ill patients when Xpert Ultra is used as the initial assay.

The observed high sensitivity of Xpert Ultra is in line with a meta-analyses, where 87.2% of TB culture positive patients were positive on Xpert Ultra test^12^ and an overall estimated pooled sensitivity of 90.9% (95% credible interval 86.2–94.7)^19^. Similar to other studies, we also observed potentially false positive Xpert Ultra results, as 4 cases were Xpert Ultra positive by culture negative. False positive Xpert Ultra results have been associated with a recent history of TB treatment^11^. In our study, the Xpert Ultra positive culture negative cases had no history of previous TB treatment. Interestingly, two of the four cases had a positive LF-LAM result, suggesting that these cases may actually be true positive instead of false positive cases of TB.

The LF-LAM assay detected 60.0% of the culture positive cases among people living with HIV, similar to findings of a study performed in four countries in sub-Saharan Africa where a 60.0% sensitivity of LF-LAM was observed among culture positive cases^20^. In contrast to studies performed during the Xpert MTB/RIF era, where addition of LF-LAM to Xpert MTB/RIF assay increased the yield^15,16^, the LF-LAM assay did not improve diagnostic yield in our cohort as all LF-LAM positive patients were also positive on Xpert Ultra. This suggests a limited role for LF-LAM in patients where Xpert Ultra can be used as the initial diagnostic as all LF-LAM negative cases would have to be assessed by Xpert Ultra due to poor LF-LAM sensitivity, and all LF-LAM positive cases would need to be assessed by Xpert Ultra for rifampicin resistance. A multicenter study in settings where Xpert Ultra is used as the initial diagnostic is needed to determine the role of LF-LAM in the diagnostic algorithm, especially for patients who cannot produce a sputum sample.

In high TB burden settings, treatment decisions often continue to rely on clinician’s judgement and chest X-ray findings, even when it has been shown that empiric treatment does not result in survival benefit^6,21^. In this study, we found that the Xpert Ultra assay detected almost all culture positive cases, questioning the value of reliance on empiric TB treatment for patients with negative Xpert Ultra results. Furthermore, adjusted analysis did not reveal six month decreased risk of death due to empirical TB treatment among Xpert Ultra negative patients, as risk of death was similar between those who did and did not receive empiric TB treatment (aOR 0.74, 95% CI: 0.1–2.7) . These results suggest that the recommendation by Kendall et al. and by Decroo et al. that clinicians should continue to prescribe TB treatment for Xpert MTB/RIF-negative patients whose clinical presentations strongly suggest pulmonary TB in order to minimize a risk of TB related mortality^6,7^ may no longer hold when Xpert Ultra is used as the initial diagnostic. All participants in whom the clinician started empiric TB treatment had been assessed by chest X-ray and had received a ‘trial’ of antibiotics (ceftriaxone and azithromycin, amoxicillin or vancomycin and doxycycline) to which they had not responded favorably. Given that empiric TB treatment in these Xpert Ultra negative patients did not decrease the risk of death, our results suggest that prescribing empiric treatment should be re-assessed as this may pose an unnecessary burden the health care system and may expose patients to unnecessary treatment. Future research should investigate which alternative diagnoses should be considered in these patient in order to develop evidence-based guidelines for the management of Xpert Ultra negative patients.

Our study had some limitations. First, we limited enrollment to hospitalized Xpert MTB/RIF-negative patients, which limits generalizability of the outpatient settings. Second, the prevalence of HIV in our cohort was relatively low (21.4%), which may limit generalizability to high HIV burden settings. Third, we excluded patients who could not provide a sputum sample. As empiric treatment and LF-LAM assay may still be of value for patients unable to produce sputum, future studies should assess the value of LF-LAM for the diagnosis of HIV positive TB in people with presumptive TB who cannot produce a sputum sample, including people with presumptive extra pulmonary TB (EPTB). Similarly, the role of empirical treatment in patients with presumptive EPTB should be investigated. Fourth, our study was not powered to assess factors associated with mortality among people with presumptive TB who had a negative Xpert Ultra assay. Consequently, the effect estimate of the association between empiric treatment and mortality in Xpert Ultra negative was imprecise. Furthermore, some participants may have had culture negative pulmonary TB although the prevalence of our cohort is likely very low as two cultures were performed in addition to Xpert MTB/RIF and Xpert Ultra. Lastly, we did not assess the presence of other respiratory pathogens to explore the cause of symptoms or death in the Xpert Ultra negative patients.

In conclusion, Xpert Ultra assay provides a sensitive, specific and rapid diagnosis of TB among presumptive pulmonary TB cases. Among patients with a negative Xpert Ultra result, the use of LF-LAM test did not yield additional cases and empiric TB treatment was not associated with mortality at six months. Future studies should be performed to establish guidelines for the management of Xpert Ultra negative patients.

## Materials and methods

### Study setting

The study was conducted at Jimma University Medical Center, a tertiary hospital located in Jimma, Oromia Region, Ethiopia. Patients are referred to the Center from health centers, district hospitals and private health facilities in Jimma Zone and district hospitals in neighboring regions, resulting in a catchment population of over 20 million. The hospital has about 800 inpatient beds and about 20,000 inpatients, 220,000 outpatient, and 15,000 emergency cases visit the hospital annually ^22^. During the study period (December 2018–July 2019), the Xpert MTB/RIF assay was used as the initial diagnostic test on sputum from presumptive pulmonary TB cases^23^. The decision to start TB treatment was made by physicians based on clinical findings (TB symptoms and their severity), response to antibiotics, chest X-ray findings (routinely performed), and HIV status and CD4 count. The Mycobacteriology Research Center of Jimma University, which is located in close proximity of the medical center, serves as the TB reference laboratory for southwest Ethiopia.

### Study population and data collection

This analysis is a secondary analysis of a cohort study that aimed to determine the role of empiric treatment among hospitalized adults (age ≥ 18 years) with symptoms of pulmonary TB (current cough, night sweats, fever, and weight loss) who could produce a sputum sample and tested negative on the Xpert MTB/RIF assay^24^. For the cohort study, all consecutive hospitalized Xpert MTB/RIF-negative patients were followed to determine if they were started on TB treatment. The first 125 patients who received empiric TB treatment and the first 125 patients not started on empiric TB treatment were offered study participation. Of those approached, two declined and three were excluded because they died prior to informed consent. A structured questionnaire was administered by trained nurses to collect demographic and clinical characteristic. Medical records of the patients were reviewed to document HIV status, CD4 count, and improvement of TB symptoms on TB treatment. Two sputum samples were collected in the first 48 h following enrolment for a single Xpert Ultra assay, a liquid culture using the Mycobacteria Growth Indicator Tube (MGIT) system and a solid culture on Lowenstein Jensen (LJ). Drug susceptibility testing for first line anti-TB drugs (rifampicin and isoniazid) was done by Line Probe assay (LPA). HIV-positive and severely ill patients (temperature > 39 °C, respiratory rate > 30 resp./min, cardiac rate > 120 bpm, or unable to walk without help)^25^ were asked to provide a 10 ml urine sample for a LF-LAM assay. All laboratory tests were performed at the Mycobacteriology Research Center of Jimma University.

### Laboratory procedures

#### Mycobacterial culture

Sputum sample decontamination and digestion, inoculation of concentrated sputum samples on LJ and MGIT media were performed using standard procedures^26^. The mycobacterial culture was classified as positive when growth was detected by LJ and/or MGIT with a positive SD Bioline TB antigen MPT64 confirmatory test and a negative blood agar test. Culture negative denotes the absence of growth on both LJ and MGIT culture or positive LJ/MGIT culture with a negative SD Bioline TB antigen MPT64 result.

#### Xpert MTB/RIF Ultra test

Xpert Ultra test was performed according to manufacturer’s instruction^27^. Briefly, 1 ml of sputum was added to 2 ml of reagent in a 15-ml Falcon tube, vortexed for at least 10 s and incubated at room temperature for 10 min. The mixture was then added to the Xpert Ultra assay cartridge and placed in the instrument. The Xpert Ultra test was repeated using the same sample in cases of an invalid result. In cases of “trace” result, a new sputum sample was collected for a second Xpert Ultra test. If the second test was positive (trace or higher), the result was considered positive for patients living with HIV and for HIV-negative patients without a history of TB treatment.

#### LF-LAM test

The urine Determine TB-LAM test (Abbott Laboratories, Lake Bluff, USA; formerly Alere Inc, Waltham, USA) was performed by applying 60μL of fresh urine to the sample pad at the bottom of the test strip using a micro-pipette. After 25 min of incubation at room temperature, the test strip was inspected visually. If any band is observed, the intensity was scored as grade 1, 2, 3 or 4 as compared with the reference card. The test was read independently by two certified laboratory professionals in blinded manner (blinded to each other and the sputum Xpert Ultra and culture results). LAM test results were classified as positive if grade ≥ 1 and as negative if no test band was observed^16^.

### Data analysis

Descriptive statistics were performed using frequencies and percentages to describe the characteristics of the study population. The yield of Xpert Ultra and LAM tests was determined by comparison to mycobacterial culture results. Mortality in the 6 months post assessment was assessed among Xpert Ultra negative participants, stratified by empiric treatment status. To identify factors associated with mortality among Xpert Ultra negative patients, we calculated the odds ratio using a logistic regression model. Data analysis was performed using R Statistical software version 3.6.1.

### Ethical considerations

Ethical clearance was obtained from the Ethical Review Board of Institute of Health of Jimma University (Ref. No: IHRPGD/397/2018). Written informed consent was obtained from all study participants. All methods were performed in accordance with the relevant guidelines and regulations. Clinicians treating study participants in whom TB was bacteriologically confirmed were notified of the result.

## Supplementary Information

Supplementary Tables.

## Data Availability

All data generated or analysed during this study are included in the article and its supplementary information files. The raw data generated in this study can be obtained by reasonable request to the corresponding author.
