# Supplementary material for: The value of lateral flow urine lipoarabinomannan assay and empirical treatment in Xpert MTB/RIF ultra negative patients with presumptive TB: a prospective cohort study
Source: Sci Rep. 2021 Dec 24;11:24428. doi: 10.1038/s41598-021-04090-1 (PMC8709852; doi:10.1038/s41598-021-04090-1)
Supplement: Supplementary file 1 — Supplementary Tables. [file 41598_2021_4090_MOESM1_ESM.docx]

**The value of lateral flow urine lipoarabinomannan assay and empirical treatment in Xpert MTB/RIF Ultra negative patients with presumptive TB: a prospective cohort study**

Wakjira Kebede^1, 2, 4*^, Gemeda Abebe^1, 2^, Esayas Kebede Gudina^3^, Annelies Van Rie^4^

**Supplementary files**

**S1 Table.** The cycle threshold (Ct) values and patient outcome at six months of the 4 Xpert Ultra positive culture negative cases.

| **Code** | **Ct value** | | | | **Outcome at six months** |
| --- | --- | --- | --- | --- | --- |
|  | **rpoB1** | **rpoB2** | **rpoB3** | **rpoB4** |  |
| Patient 1 | 30.8 | 29.6 | 31.8 | 33.8 | Survived |
| Patient 2 | 30.4 | 29.8 | 31.4 | 32.3 | Survived |
| Patient 3 | 29.2 | 28.8 | 30.7 | 32.4 | Survived |
| Patient 4 | 29.7 | 29.0 | 31.3 | 33.5 | Survived |
